# Dissecting the cell of origin of aberrant SALL4 expression in myelodysplastic syndrome

**DOI:** 10.1002/ctm2.1327

**Published:** 2023-07-27

**Authors:** Hiro Tatetsu, Miho Watanabe, Jun Liu, Kenji Tokunaga, Eisaku Iwanaga, Yoshihiro Komohara, Emily Thrash, Mahmoud A. Bassal, Masao Matsuoka, Daniel G. Tenen, Li Chai

**Affiliations:** ^1^ Department of Hematology Rheumatology and Infectious Diseases Faculty of Life Sciences Kumamoto University Kumamoto Japan; ^2^ Department of Pathology Brigham and Women's Hospital Harvard Medical School Boston Massachusetts USA; ^3^ Department of Cell Pathology Faculty of Life Sciences Kumamoto University Kumamoto Japan; ^4^ FLUIDIGM Fluidigm Canada Inc. Markham Ontario Canada; ^5^ Harvard Stem Cell Institute Boston Massachusetts USA; ^6^ Cancer Science Institute of Singapore Singapore

Dear Editor,

Despite the increased reports on oncofetal protein Spalt like transcription factor 4 (SALL4) in myelodysplastic syndrome (MDS), the cellular identity of its aberrant expression in MDS patients remains unknown. Uncovering the answer to this question has important implications for furthering our understanding of the pathogenesis of MDS, as well as for the development of therapeutic targeting of SALL4 in MDS. Our study is the first to characterize aberrant SALL4 expression in MDS patient samples at a single cell level.

Myelodysplastic Syndromes (MDS) are a heterogeneous group of diseases characterized by cytologic dysplasia and cytopenias resulting from ineffective hematopoiesis.^1^ Up to 30% of patients with MDS may transform to acute myeloid leukemia (AML). Despite the well‐characterized cytogenetic changes and gene mutations associated with MDS, the pathogenesis of the disease remains incompletely understood, which is a major barrier to developing effective therapeutic strategies.^1^ SALL4, a transcription factor important for development and embryonic stem cell properties,^2^ is aberrantly expressed in various cancers^3^, ^4^ and is a known leukemic oncogenic driver.^5^, ^6^, ^7^ Its expression level serves as a prognostic biomarker for MDS at the time of diagnosis.^8^ We recently showed that SALL4 up‐regulation following hypomethylating agent treatment in MDS patients correlates with poor outcomes.^9^ To further investigate the cellular identity of bone marrow cells with aberrant SALL4 expression in MDS patients, we utilized mass cytometry (CyTOF) to analyze MDS bone marrow cells in comparison to controls. Additionally, to address the relationship between SALL4 expression and somatic gene mutations, which is important for risk stratification and identification of disease drivers, we performed paired bone marrow Whole Exome Sequencing (WES) on these samples. DNA from bone marrow (BM) mononuclear cells from MDS and control samples (lymphoma without bone marrow infiltration) were extracted according to the manufacturer's recommendations (Table S1).

We first examined SALL4 mRNA expression in MDS CD34+ cells by analyzing MDS expression profiles from the public database GSE19429, which contained 183 patients with MDS and 17 healthy controls. We observed that SALL4 expression in MDS CD34 cells was higher than that of controls, with the highest expression in refractory anemia with excess blasts type 2 (RAEB2) (*p* ≤ .05) (Figure 1A,B). To further investigate the protein expression pattern of SALL4 in MDS patients’ BM, we performed CyTOF experiments, which included 22 surface markers of cell surface proteins and 5 markers of intracellular signaling with single‐cellular resolution (Table S2). We validated the CyTOF method in cell lines, using published gating methods (Figures S1 and S2). Our results showed that MDS patients had aberrant SALL4 protein expression in hematopoietic stem cells (HSC) (*p* ≤ .05), hematopoietic progenitor cells (HPC) (*p* ≤ .05) and myeloid lineage cells (*p* ≤ .05) when compared to controls (Figure 1C and Figure S2). Understanding the cell of origin with aberrant SALL4 expression has important implications for understanding MDS pathogenesis, as well as SALL4 therapeutic targeting in myeloid neoplasms. Recent advances in the understanding of myeloid neoplasm pathogenesis and refined analysis of AML bone marrow cell‐of‐origin studies have led to the design of therapeutic targeting of leukemia stem cells as a more promising approach toward a cure.

**FIGURE 1:**
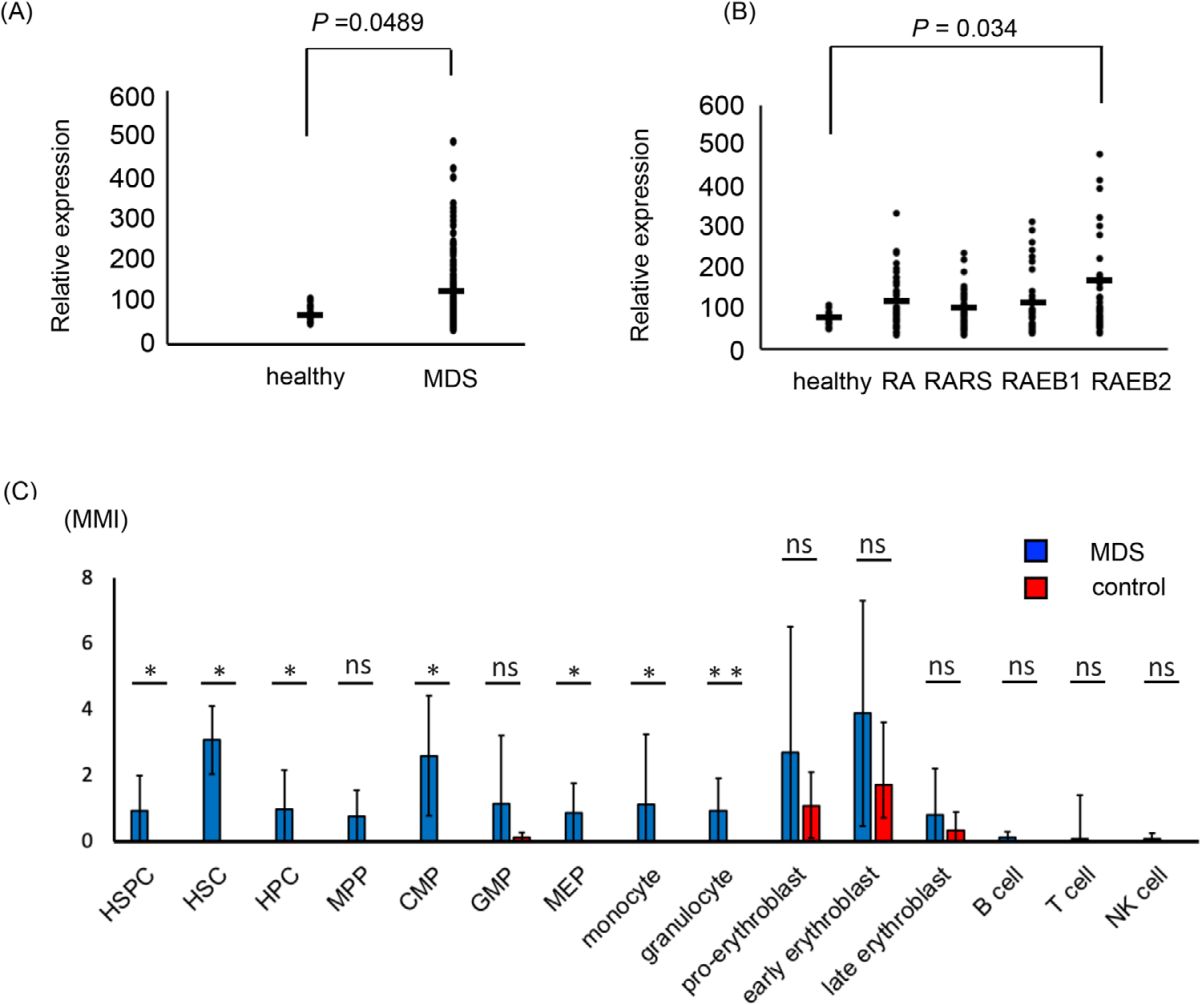
SALL4 RNA and protein were expressed in MDS bone marrow cells. (A) SALL4 expression in MDS CD34 positive cells from the public database GSE19429. (B) SALL4 expression in CD34 positive cells in MDS subtype from the public database GSE19429. RA, refractory anemia; RARS, refractory anemia with ring sideroblasts; RAEB1, refractory anemia with excess blasts1; RAEB2, refractory anemia with ring sideroblasts2. (C) MMI of SALL4 in bone marrow cells on CyTOF (MDS patients, *n* = 10, control BM, *n* = 5, error bars indicate standard deviation). Data analysis used Mann–Whitney *U* test. *p ≤ .05; **p ≤ .01. ns, not significant; HSPC, hematopoietic stem and progenitor cell; HSC, Hematopoietic stem cell; HPC, hematopoietic progenitor cell; MPP, multipotent progenitors, CMP; common myeloid progenitor; GMP granulocyte‐monocyte progenitor; MEP, megakaryocyte‐erythrocyte progenitor; NK cell, natural killer cell; MMI, median metal intensity.

Next, we investigated the relationship between SALL4 expression with p53, ki67, c‐myc or phosphorylated AKT (pAKT), which have important roles in leukemia or MDS pathogenesis,^1^ on single cell level. SALL4 expressing cells expressed p53 (*p* = .01) in hematopoietic stem/progenitor (HSPC) (Figure 2A) and showed a trend of a higher level of pAKT and ki67.

**FIGURE 2:**
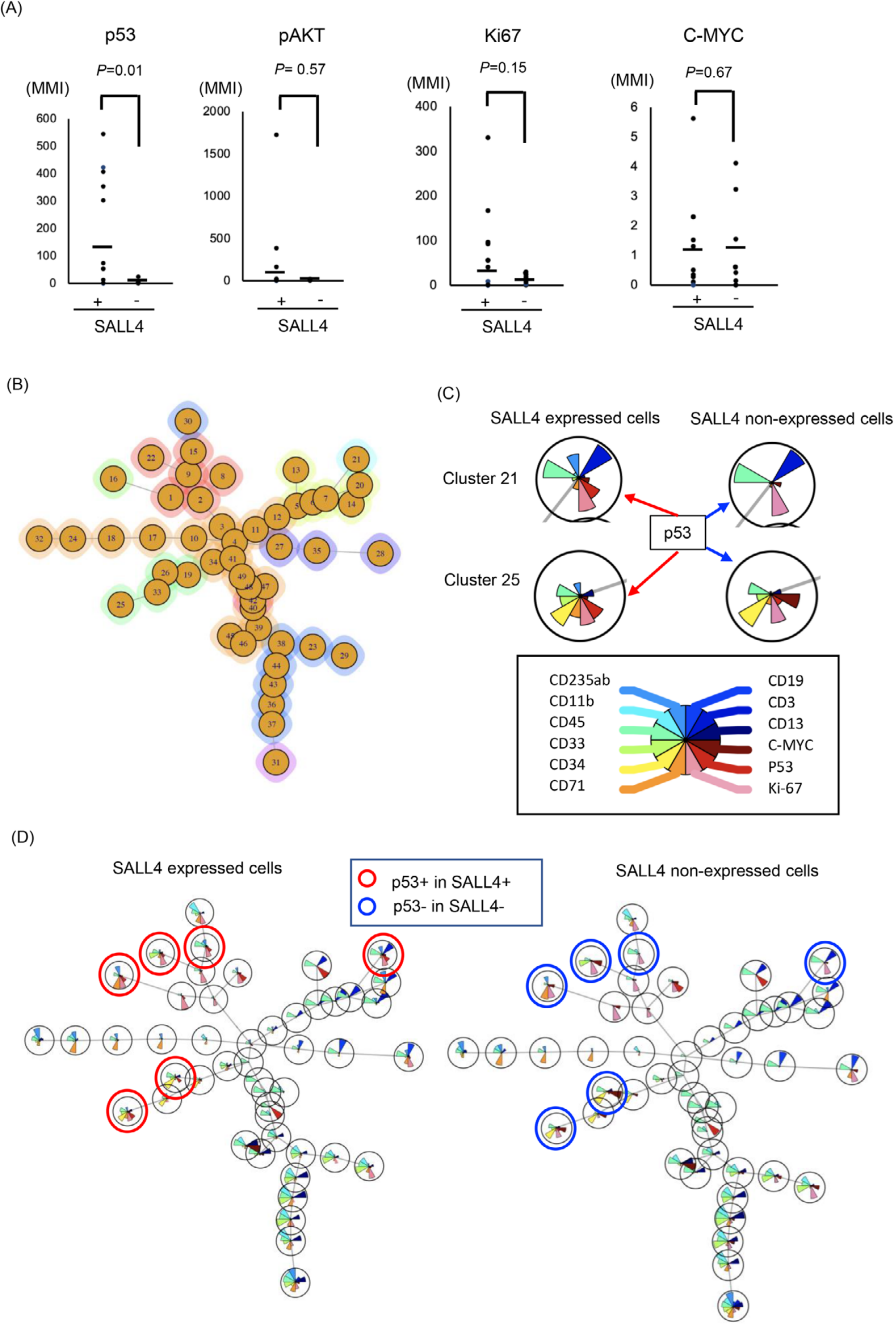
ViSNE analysis on SALL4 expressing cells in MDS samples. (A) P53, ki67, c‐myc or pAKT expression of SALL4 expressing cells and SALL4 non‐expressing cells. (B) Cells were clustered into 49 groups on FlowSOM analysis. (C) The example of FlowSOM via viSNE analysis based on the distribution of SALL4, p53, Ki67 and major lineage markers (CD235ab, CD11b, CD45, CD33, CD34, CD71, CD19, CD3, CD11b) on SALL4 expressing cells (left side) and SALL4 non‐expressing cells (right side) on cluster 21 and 25. The brown fan shape indicates p53. (D) FlowSOM via viSNE analysis on SALL4 expressing cells (left panel) and SALL4 non‐expressing cells (right panel). The pie‐chart visualizes the relative expression. The red circle indicated both p53 and SALL4 expressed cells. The blue circle indicated only p53 expressed, and SALL4 non‐expressed cells.

Next, FlowSOM via viSNE analysis was performed on MDS bone marrow cells to understand the expression pattern of SALL4 and p53 and to investigate the relationship between them. This analysis distinguished 49 clusters (Figure 2B & C). SALL4 expressing clusters tended to express p53, whereas SALL4 non‐expressing cells did not (Figure 2C & D). Minimal spanning trees (MSTs) analysis by FlowSOM (Figure 3A) distinguished 10 meta‐clusters (Figure 3B, Figure S3A), with SALL4+p53+ cells mainly present in meta‐cluster 9 (Figure 3C, Figures S3 and S4).

**FIGURE 3:**
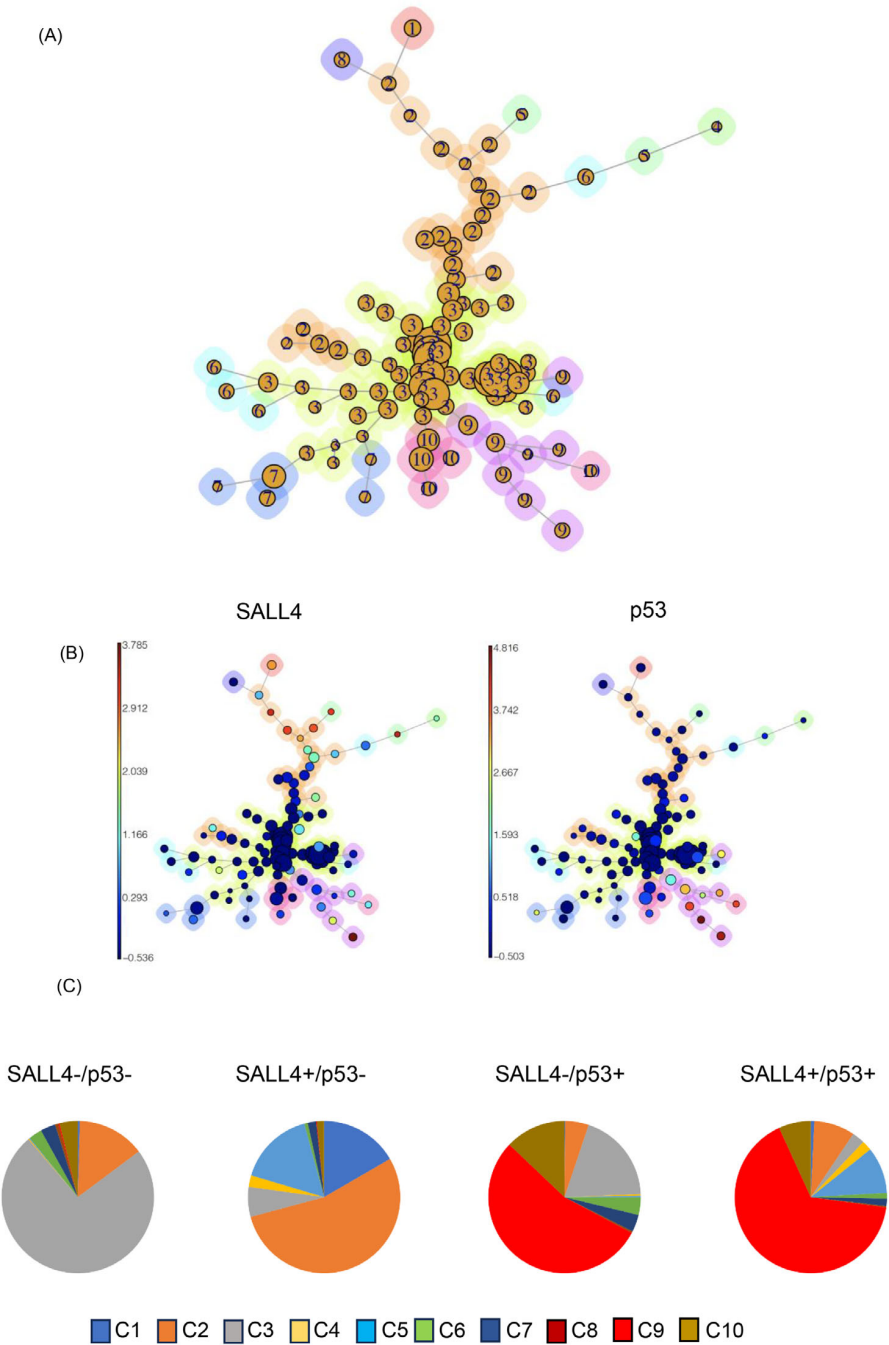
Examination of SALL4 and p53 expression in MDS bone marrow cells. (A) Cells were clustered into 12 nodes and depicted as a minimal spanning tree, to the ten identified clusters on FlowSOM analysis. (B) Aggregated events of SALL4 (left) and p53 (right) on FlowSOM analysis. (C) The pie chart showing percentage of SALL4‐p53‐ cells, SALL4+p53‐ cells, SALL4‐p53+ cells and SALL4+p53+ cells in each cluster.

Previously, the relationship between SALL4 expression and somatic gene mutations (e.g., TP53), which are important for risk stratification and identification of disease drivers, had not been well explored in MDS patients. Using concomitant WES data, we were able to gain further insights into the relationship between the TP53 mutation status and SALL4/TP53 co‐expression in MDS patients. Notably, both patients number 1 and number 2, who had the highest fractions of SALL4/TP53 double positive cells, harbored pathogenic loss‐of‐function TP53 mutations in the DNA binding domain (Table 1). For patient NO.7, the p.V173* mutation was a frameshift variant that likely resulted in nonsense mediated decay and reduced/absent TP53 protein expression. Thus, it was not surprising that a significantly smaller fraction of SALL4/TP53 positive cells was detected in patient NO.7 despite the presence of a deleterious TP53 mutation. Using the TCGA AML dataset, we found that TP53 mutations are enriched in patients having high SALL4 expression (Figure S5). The mechanistic interplay between SALL4 and TP53 requires further investigation.

**TABLE 1:** SALL4 & p53 double positive cells and karyotypes & mutations in MDS.

Sample number	Cytogenetics	Mutations	% of SALL4 & p53 DP cells (C9)	TP53 mutations, if present	VAF	Variant description
1	44,X,‐Y,−5,−7,8,−9,add(17)(p11.2),−18,−20 +mar1,mar2,mar3[12]/46,XY[3]	GNAS, GATA2, KMT2C, TP53	56.3	c.476C > T	.58	Missense variant in DNA binding region
2	46,XY,−6,−17,−19,−20,+mar1, +,mar2[15]/46,XY[5]	KMT2C, TP53	40	c.782+1G > C	.46	Intronic splice site variant, DNA binding domain
3	46,XX,+1,der(1;7)(q10;p10){11]/45, idem, −18[4]/46,XX[5]	KMT2C	22.5	–	–	–
4	46,XX,t(3;21)(q26.2;q22.1)[20]	CALR, GATA2, KIT	14.3	–	–	–
5	46,XY,der(1;7)(q10;p10)[1]/46,XY[19]	ETNK1, FLT3, KMT2C, TET2, ZRSR2,	13.02	–	–	–
6	46,XY,+1,der(1;7)(q10;p10)[6]/idem,del(20)(q1?)[11]/46,XY[3]	ETNK1, EZH2, GATA2, PTPN11, SETBP1, TERT	9.54	–	–	–
7	42,XY,−4,−5,−7,add(7),9,add(12)(p11.2),add(12)(q24.1),add(13)(p11.2),−17,−18,−20,+mar1,+mar2,+mar3[15]/46,XY[3]	MYBL2, TP53	6.74	p.V173*	.45	Frameshift variant affecting DNA binding domain
8	46,XX, del(20)(q1?)[5]/46,XX[15]	DNMT3A, GATA2, GNAS, SF3B1	5.33	–	–	–
9	46,XY	DNMT3A, ETNK1, SF3B1, TET2	3.68	–	–	–
10	46,XY	PRPF8, RUNX1, SF3B1, TET2	2.43	–	–	–

Abbreviations: DP, double positive; VAF, variant allelic frequency.

## CONCLUSION

The identification of MDS‐clones in MDS patients that may progress to AML is of great importance for the treatment of this disease. While SALL4, when constitutively expressed, has been shown to cause MDS/AML in a murine model, the cellular identity of aberrant SALL4 in human MDS remains unknown. Utilizing state‐of‐the‐art single cell mass cytometry and paired Whole Exome Sequencing, our study has, for the first time, identified aberrant expression of SALL4 in hematopoietic stem/progenitor cells and myeloid lineage cells in human MDS patient bone marrow samples. Additionally, we observed a significant SALL4+p53+ cluster in the MDS bone marrow, particularly prominent in patients with pathogenic TP53 mutations. Our study provides a foundation for future investigations into the mechanisms and therapeutic targeting of SALL4 in the pathologic lineages of MDS, with potential prognostic and therapeutic implications.

## CONFLICT OF INTEREST STATEMENT

H.T. has received honoraria from Meiji Seika Pharma, Takeda Pharmaceutical, Novartis International, Bristol Myers Squibb, Chugai Pharmaceutical, Eisai, Ono Pharmaceutical, SymBio Pharmaceuticals Limited and patents, and royalties from Mesoblast. L.C and D.G.T also receive royalties from Mesoblast. Other authors have no conflict of interest to disclose.

## Supporting information

Supporting Information

Supporting Information

Supporting Information
