# Supplementary material for: Dissecting the cell of origin of aberrant SALL4 expression in myelodysplastic syndrome
Source: Clin Transl Med. 2023 Jul 27;13(8):e1327. doi: 10.1002/ctm2.1327 (PMC10374880; doi:10.1002/ctm2.1327)
Supplement: Supplementary file 2 — Supporting Information [file CTM2-13-e1327-s002.docx]

**Supplementary Figure 1. Detection of SALL4 protein in leukemia cell lines via CyTOF**

SALL4 was expressed in NB4, a human promyelocytic leukemia cell line and was not expressed in MT4, a human adult T-cell leukemia cell line.

**Supplementary Figure 2. Representative CyTOF data of SALL4 expression in MDS patients and controls**

The upper two panels show SALL4 expression in HSC, granulocyte, B cell and erythroid cells in two representative MDS patients (No.2 and No.3). The lower two panels show those in two representative control patients. Abbreviations: HSPC, hematopoietic stem/progenitor cells.


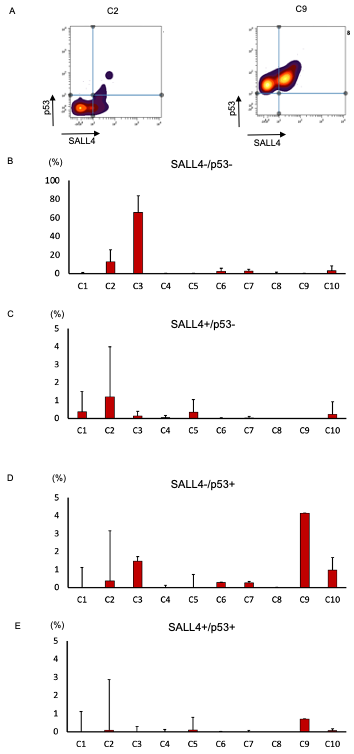


**Supplementary Figure 3. Further analysis on SALL4 and/or p53 expressing cells on MDS samples**

(A) The panel shows a representative pattern of Cluster2 (C2) and Cluster9 (C9) on CyTOF. (B) The percentage of SALL4-p53- cells. (C) The percentage of SALL4+p53- cells (D) The percentage of SALL4-p53+ cells. (E) The percentage of SALL4+p53+ cells. Error bars indicate standard deviation.

**Supplementary Figure 4. Aggregated events of surface markers**

Aggregated events of CD45, SALL4, p53, CD34, CD38, CD33 CD13, CD11b, CD235ab, CD71, CD3 and CD19.

**SALL4 expression TP53 mutation**


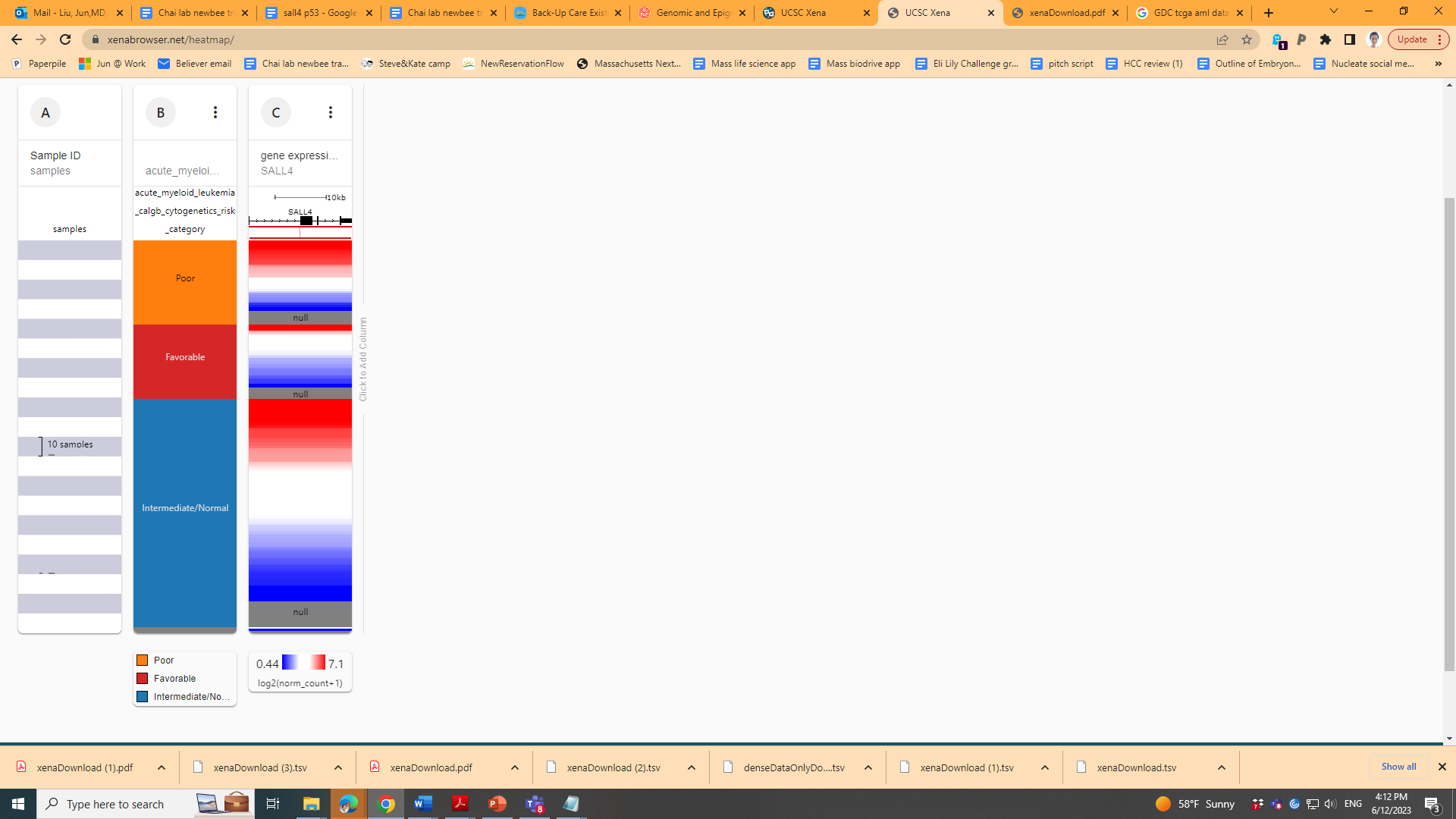

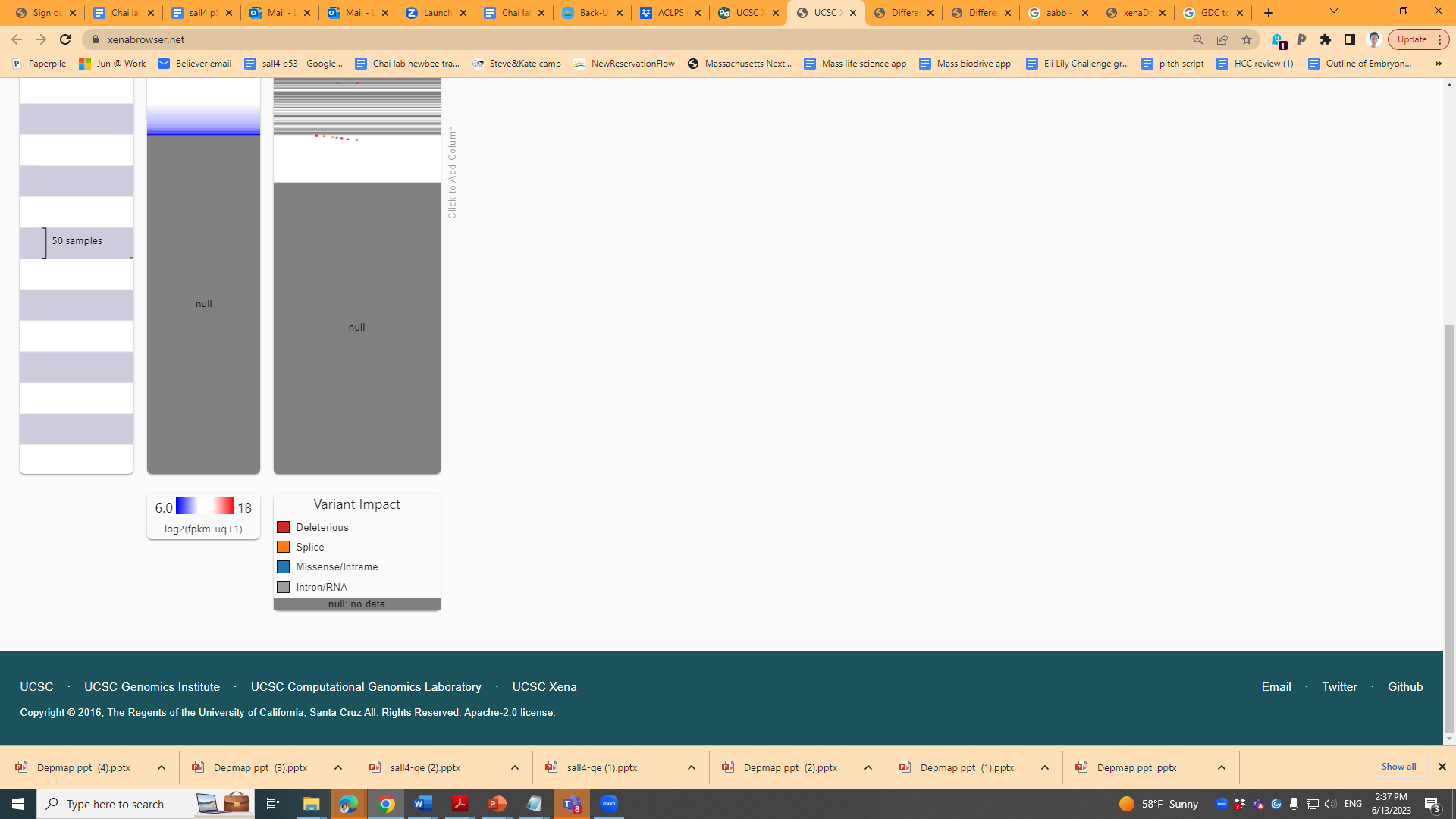

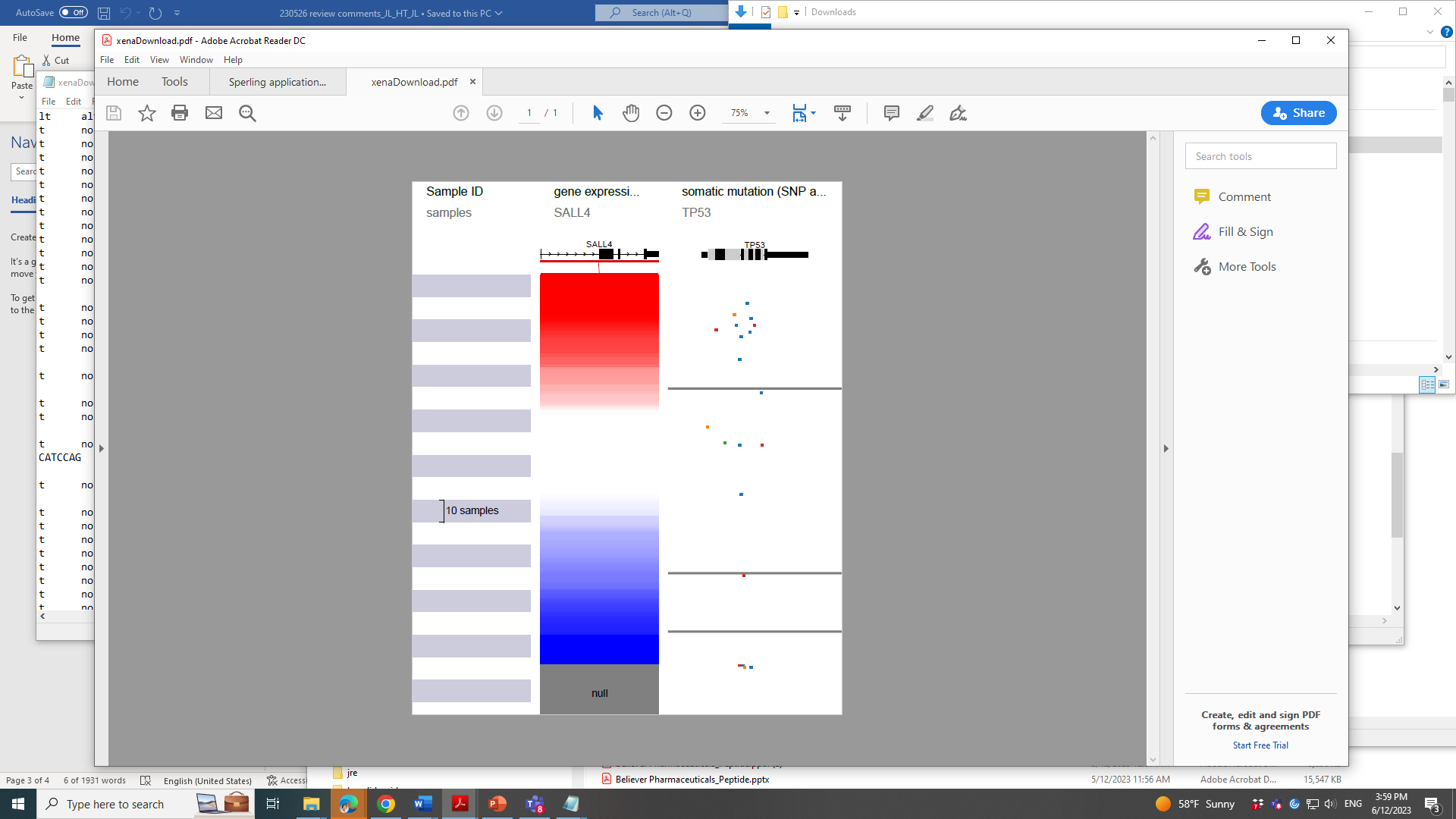


**Supplementary Figure 5. TP53 mutations are enriched in patients having high SALL4 expression.** The Cancer Genome Atlas (TCGA) AML dataset contains gene expression profiles (mRNA) and mutation status of all the patient samples. TP53 mutations are color-coded by variant impacts. The three grey bars represent three samples without TP53 mutation data.
