# Supplementary material for: Dissecting the cell of origin of aberrant SALL4 expression in myelodysplastic syndrome
Source: Clin Transl Med. 2023 Jul 27;13(8):e1327. doi: 10.1002/ctm2.1327 (PMC10374880; doi:10.1002/ctm2.1327)
Supplement: Supplementary file 3 — Supporting Information [file CTM2-13-e1327-s001.docx]

**Supplementary Materials and Methods**

**Study approval**

This research was approved by the Ethics Committee of Kumamoto University (approval genome No. 297). DNA from bone marrow mononuclear cells from 10 MDS was extracted using the according to the manufacturer’s recommendations (**Suppl Table 1)**. We performed CyTOF^®^ (Fluidigm^®^) and WES (Novogene Bioinformatics Institute, Beijing, China) for MDS patients and CyTOF for 5 lymphoma patients, on which the invasion of bone marrow was ruled out, as the controls.

**Metal-tagged monoclonal antibodies**

A panel of 27 metal-tagged monoclonal antibodies was used for analysis of the patient's bone marrow mononuclear cells. A detailed listing of antibodies and corresponding metal tags is provided in **Suppl Table 2**. Antibodies were purchased either already metal-tagged (Fluidigm®) or in purified form. They were labeled using the Maxpar® X8 Antibody Labeling Kit (Fluidigm) according to the manufacture’s recommended protocol, titrated, and were diluted to 0.5 mg/ml in Antibody Stabilizer (CANDOR Bioscience) for long-term storage at 4℃.

**Antibody staining for mass cytometry**

Bone marrow mononuclear cells were thawed at 37℃, resuspended in 10% FBS in RPMI1640 and washed twice with PBS (without calcium or magnesium). Bone marrow blood cells were then transferred to 1.5ml tube and were stained 5 minutes in PBS supplemented with 0.2μM Cisplatin Cell-ID™ (Fluidigm, San Francisco, CA). Cells were suspended and washed with Maxpar® cell staining buffer (Fluidigm). Then cells were resuspended in Maxpar® cell staining buffer (Fluidigm) and blocked with Human TruStain FcX™(Biolegend) for 10 minutes at room temperature. Cells were incubated with an antibody targeting cell surface markers for 30 minutes at room temperature and then washed twice with cell staining buffer. After washing, cells were fixed and permeabilized for 30 minutes on ice using eBioscience FoxP3 fix/perm (Thermo Fisher Scientific, Waltham, MA, USA). Fixed/permeabilized cells were washed twice with 1×working solution of permeabilization buffer(eBiosciences) and incubated with all antibodies targeting intracellular antigens for 30 minutes at room temperature. After staining with intracellular antibodies, cells were washed twice with 1× working solution of permeabilization buffer and incubated with Cell-ID Ir DNA intercalator (Fluidigm) over night at 4℃. On the next day, prior to mass cytometry analysis, cells were washed twice with Maxpar® cell staining buffer (Fluidigm) and resuspended in water containing EQ™ Four Element Calibration Beads (Fluidigm). Samples were acquired on a Helios™ CyTOF System (Fluidigm).

**Mass cytometry analysis**

Cells were analyzed on a mass cytometry (Helios, CyTOF System) (Fluidigm) at an event rate of approximately 300 cells/second. The resulting data were analyzed with software available through Cytobank ([www.cytobank.org](http://www.cytobank.org)). For mass cytometry data, all parameters except time and event length were displayed with an arcsinh transformation and a scale augment of 5 ranging from -5 to 12000. Event length and time were displayed on linear scales. 191Ir and 193Ir DNA intercalator and 140Ce beads were used to discern intact singlets from debris and cell aggregates. Then live single cells were selected by applying a gate on 193Ir DNA vs. 198Cisplatin. All gating and extraction of median expression levels were performed using Cytobank. To interpret high-dimensional single-cell data that were produced by mass cytometry, we used a visualization tool based on the viSNE and FlowSOM algorithm, which allows visualization of high-dimensional cytometry data on a 2-dimensional map at single-cell resolution and preserves the nonlinearity^1^ ^2^. FlowSOM is an algorithm that allows us to visualize both manual gatings and automated clusterings to divide cell types into clusters and meta-clusters, based upon the similarity of cell-specific marker expression profiles using pie charts^2^. Immunophenotypic subsets on the basis of standard surface markers were analyzed based on the European LeukemiaNet recommendation and the related publications ^3^ ^4^ ^5^ **(Suppl Table 2)**. All gating and extraction of median expression levels were performed using Cytobank. CyTOF data analysis was performed using viSNE and FlowSOM, which is an advanced clustering analysis software in Cytobank™ (Beckman Coulter Life Sciences, Indianapolis, IN).

Cell subsets defined as HSPC (haematopoietic progenitor stem cells): Lin-CD34+, HSC (haematopoietic stem cells): Lin−CD38−CD34+, MPP (multipotent progenitors): Lin−CD34+CD38+CD90−CD45RA-CD49f−, CMP (common myeloid progenitors): Lin−CD38+CD34+ CD90−CD123+CD45RA−: GMP (granulocyte macrophage progenitors): Lin−CD38+ CD90−CD34+CD123+CD45RA+ and MEP (megakaryocyte-erythroid progenitors): Lin−CD38+ CD90−CD34+CD123+CD45RA+cells. Lineage negative cells were defined as following CD11b, CD38, CD3, CD7, CD19, CD71, CD235, and CD33. Monocyte Lineage cells were defined as following criteria: not CD34+CD38 low, CD33 positive, HLA-DR positive, not CD123 bright and CD33 low, CD3 negative, not CD19+, not CD45 high and CD7 high, not brightly CD38 positive, not CD71 high or CD235 high. Granulocyte Lineage cells were defined as following criteria: not CD71 high or CD235 high, CD3 negative, not CD19+, not brightly CD38 positive. Erythroid Lineage cells were defined as following criteria: not CD34+CD38 low, CD3 negative, not CD19+. Within this population, CD71 bright CD235 low cells were defined as pro-erythroblasts, CD71 bright CD235 positive cells were defined as early erythroblasts, and late erythroblasts were defined as CD235 positive CD71 mid and CD45 negative. B cell Lineage cells were defined as following criteria: CD33 low, CD3 negative, not CD71high or CD235 high, CD11b low. CD3 positive was defined as T cells. NK cells were defined as following: positive for CD7 and CD45, expression of CD7 in the absence of CD3, and lack of CD34 expression.

**Data mining**

Publicly available database GSE19429 consists of gene expression profiles from MDS patients of various subtypes. Gene expression profiles were generated using Affymetrix Human Genome U133 Plus 2.0 Array. The serial matrix files were downloaded from Gene Expression Omnibus.

We used the Cancer Genome Atlas (TCGA) AML dataset to confirm the relationship between SALL4 expression and TP53 mutations. Processed data were queried via <https://xena.ucsc.edu/compare-tissue/>.

**Whole Exome Sequencing**

The bone marrow mononuclear cells were collected using Ficoll-Paque™ PREMIUM (Cytiva, Tokyo, Japan). Novogene (Beijing, China) performed whole-exome sequencing (WES) including exome capture, high throughput sequencing, and common filtering by purified genomic DNA. Raw reads were aligned to the hg38 genome using BWA ^6^. Single nucleotide variants (SNV), insertions and deletions (INDEL) were called using GATK v4.0. ANNOVAR and MutationTaster were used to annotate variants ^7^ ^8^. To exclude common single nucleotide polymorphisms (SNP) in tumor-only samples, pre-filtering was performed according to the following criteria: 1) ExAC_ALL < 0.01, 2) MutationTaster flag = D (disease causing) or A (disease causing automatic). Subsequent analyses were conducted on the recurrently-mutated 62 genes in MDS, which were selected from previous publications^9,10^, listed in **Suppl Table 3.**

**Statistical analysis**

All statistical analyses were performed with Excel and EZR (Saitama Medical Center, Jichi Medical University, Saitama, Japan), which is a graphical user interface for R (The R Foundation for Statistical Computing, Vienna, Austria). More precisely, it is a modified version of R commander designed to add statistical functions frequently used in biostatistics^11^. Statistical significances of two group comparisons were determined using Mann–Whitney U test. Multiple comparisons were performed by one-way ANOVA with Tukey correction. The minimum significance level was set at *P* < 0.05. Asterisks indicate the statistical significance as follows: **P* ≤ .05; ***P* ≤ .01.

**References**

1. Amir el AD, Davis KL, Tadmor MD, et al. viSNE enables visualization of high dimensional single-cell data and reveals phenotypic heterogeneity of leukemia. *Nat Biotechnol*. 2013;31(6):545-552.

2. Van Gassen S, Callebaut B, Van Helden MJ, et al. FlowSOM: Using self-organizing maps for visualization and interpretation of cytometry data. *Cytometry A*. 2015;87(7):636-645.

3. Della Porta MG, Picone C, Pascutto C, et al. Multicenter validation of a reproducible flow cytometric score for the diagnosis of low-grade myelodysplastic syndromes: results of a European LeukemiaNET study. *Haematologica*. 2012;97(8):1209-1217.

4. Westers TM, Ireland R, Kern W, et al. Standardization of flow cytometry in myelodysplastic syndromes: a report from an international consortium and the European LeukemiaNet Working Group. *Leukemia*. 2012;26(7):1730-1741.

5. Behbehani GK, Finck R, Samusik N, et al. Profiling myelodysplastic syndromes by mass cytometry demonstrates abnormal progenitor cell phenotype and differentiation. *Cytometry B Clin Cytom*. 2020;98(2):131-145.

6. Li H, Durbin R. Fast and accurate short read alignment with Burrows-Wheeler transform. *Bioinformatics*. 2009;25(14):1754-1760.

7. DePristo MA, Banks E, Poplin R, et al. A framework for variation discovery and genotyping using next-generation DNA sequencing data. *Nat Genet*. 2011;43(5):491-498.

8. Schwarz JM, Rodelsperger C, Schuelke M, Seelow D. MutationTaster evaluates disease-causing potential of sequence alterations. *Nat Methods*. 2010;7(8):575-576.

9. Sperling AS, Gibson CJ, Ebert BL. The genetics of myelodysplastic syndrome: from clonal haematopoiesis to secondary leukaemia. *Nat Rev Cancer*. 2017;17(1):5-19.

10. Makishima H. [Sequential acquisition of mutations in myelodysplastic syndromes]. *Rinsho Ketsueki*. 2017;58(10):1828-1837.

11. Kanda Y. Investigation of the freely available easy-to-use software 'EZR' for medical statistics. *Bone Marrow Transplant*. 2013;48(3):452-458.
